# Pre-Existing Diabetes Mellitus, Hypertension and KidneyDisease as Risk Factors of Pre-Eclampsia: A Disease of Theories and Its Association with Genetic Polymorphism

**DOI:** 10.3390/ijerph192416690

**Published:** 2022-12-12

**Authors:** Abdullah Salah Alanazi, Francis Victor, Kanwal Rehman, Yusra Habib Khan, Ismaeel Yunusa, Abdulaziz Ibrahim Alzarea, Muhammad Sajid Hamid Akash, Tauqeer Hussain Mallhi

**Affiliations:** 1 Department of Clinical Pharmacy, College of Pharmacy, Jouf University, Sakaka 72388, Saudi Arabia; 2 Department of Pharmacy, University of Chenab, Gujrat 50700, Pakistan; 3 Department of Pharmacy, The Women University, Multan 66000, Pakistan; 4 College of Pharmacy, University of South Carolina, Columbia, SC 29208, USA; 5 Department of Pharmaceutical Chemistry, Government College University, Faisalabad 38000, Pakistan

**Keywords:** pathogenesis, pre-eclampsia, diabetes, proinflammatory mediators, risk factors, antiphospholipid syndrome

## Abstract

Pre-existing diabetes, hypertension and kidney disorders are prominent risk factors of pre-eclampsia (PE). It is a multifactorial pregnancy disorder associated with high blood pressure, proteinuria, and multiorgan failure, which develops after the 20th week of pregnancy. It is one of the most feared pregnancy disorders, as it consumes thousands of fetomaternal lives per annum. According to clinical and pathological studies, the placenta appears to be a key player in the pathogenesis of PE; however, the exact origin of this disorder is still under debate. Defective placentation and angiogenesis are the hallmarks of PE progression. This angiogenic imbalance, together with maternal susceptibility, might determine the severity and clinical presentation of PE. This article comprehensively examines the mechanisms of pathogenesis of PE and current evidence of the factors involved in its progression. Finally, this article will explore the genetic association of PE, various candidate genes, their proposed mechanisms and variants involved in its pathogenesis.

## 1. Introduction

Pre-eclampsia (PE), the “disease of theories”, is a multifactorial pregnancy disorder associated with high blood pressure and the presence of protein in the urine, probably occurring after 20 weeks of pregnancy, which if left untreated leads to organ failure [1,2]. Pre-existing or gestational diabetes, hypertension and kidney disorders are some important contributors to PE. If left untreated, it leads to severe dysfunctions such as hemolysis, shortness of breath, pulmonary edema, liver and kidney dysfunction, blurred vision, restricted placental growth and ultimately eclampsia [3]. Furthermore, Kuklina and Ayala et al. unraveled the association of PE with future cardiovascular and renal disorders, particularly in people with severe pre-eclampsia [4]. Almost 2–7% of pregnancies are at risk of developing PE. PE has a high motility and morbidity rate, with 70,000 plus maternal and around 0.5 million fetal deaths per annum [4,5,6]. The concept of PE has changed with time. Previously, it was thought to be related to kidney disorders, such as lupus nephritis [7]. However, it is now associated with toxemia caused by certain circulating toxins (antiangiogenic and proinflammatory proteins) in the blood. This concept was transformed when some antiangiogenic factors were detected in the urine of PE patients [8]. Extensive studies performed on PE have proposed numerous mechanisms of its pathogenesis, for instance, spiral arteries, endothelial dysfunction, oxidative stress, hypoxia, decreased placental perfusion and altered immune response. [9]. Furthermore, many maternal, fetal, environmental and genetic risk factors are involved in the development of PE [10].

With advancements in technology and understanding, the genetic research of PE has captured the attention of the research community. Around 70 candidate genes were found to be associated with the pathogenesis of PE, and research is continuing to unravel the genetic components of pre-eclampsia [11]. The focus of this review is to offer a comprehensive overview of the clinical markers, pathogenesis, risk factors and genetic associations of PE.

## 2. Clinical Manifestations of Pre-Eclampsia

According to ACOG (American College of Obstetrics and Gynecology), PE is defined as a hypertensive disorder, occurring after 20 weeks of gestation with the presence of protein in the urine of patients. Most patients usually develop clinical symptoms of pre-eclampsia, such as low platelets and liver dysfunction, before showing a detectable concentration of protein in the urine. These are the results of delayed diagnosis, which can be dangerous for the patients [12,13]. In 2013, the ACOG task force redefined pre-eclampsia as “a heterogenous hypertensive disorder”. Proteinuria was excluded as the sole factor for diagnosis of PE. These biomarkers and features are demonstrated in Table 1 and were also confirmed in recent updates [14].

## 3. Pathogenesis of Pre-Eclampsia

Extensive animal models and human studies have been conducted on investigate the pathogenesis of PE. These studies demonstrated the progression of PE in two stages; the first stage is “abnormal placental growth”, developing in the first trimester. The second stage, also known as “maternal syndrome”, occurs in the second and third trimesters, and is usually characterized by an excessive antiangiogenic factor [15].

## 4. Stage 1 of Pre-Eclampsia Pathogenesis

**Figure 1:**
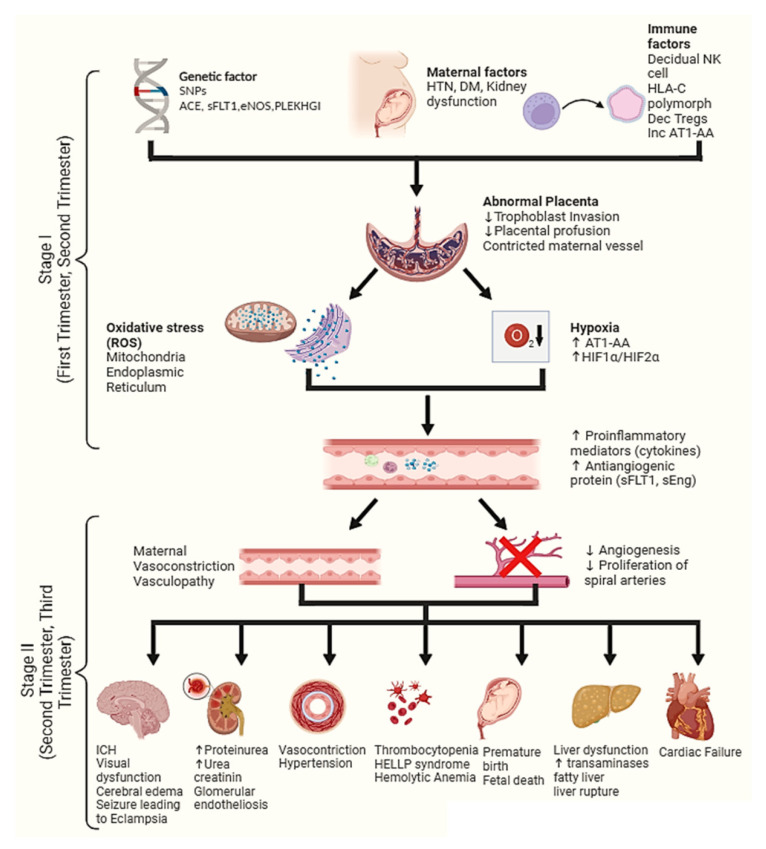
Stages of the pathogenesis of pre-eclampsia. Stage I shows factors such as genetic, maternal, and immune factors that are responsible for abnormal placental formation. This leads to hypoxia and high oxidative stress, which causes the release of proinflammatory mediators and antiangiogenic factors, causing multiple organ failure, eclampsia, premature birth, or fetal death. In stage II, maternal vasoconstriction, vasculopathy, decreased angiogenesis, and a decrease in spiral artery proliferation led to multiorgan failure. *Abbreviations*|SNP: single-nucleotide polymorphism; sFLT1: soluble FMS-like tyrosine kinase-1; eNOS: endothelial nitric acid synthase; ACE: angiotensin-converting enzymes; PLEKHG1: pleckstrin homology and RHOGEF domain containing G1; HTN: hypertension, DM: diabetes mellitus; NK cell: natural killer cell; HLA-C: human leukocyte antigen-C, AT1-AA: angiotensin I and II autoantibodies; Tregs: regulatory T cell; HIF1α: hypoxia-inducible factor 1 alpha; HIF2α: hypoxia-inducible factor 2 alpha; ROS: reactive oxygen species; ICH: intracerebral hemorrhage; sEng: endothelin-1.

Some researchers consider stage 1 (abnormal placental growth) to be controversial. However, animal studies have shown the abnormal placenta leads to uteroplacental ischemia, which causes hypertension, and multiorgan failure proceeding to stage 2 (maternal syndrome) [16,17]. Numerous theories have been proposed by different researchers that contribute to the placental dysfunction in stage 1, for instance, hypoxia, oxidative stress, and natural killer cell (NK) abnormalities, as demonstrated in Figure 1. However, none of these theories have conclusive evidence in human models [16,17,18].

In normal placentation, the cytotrophoblast cells drift towards the uterine spiral arteries of the mother, providing nutrition to the fetus by forming vascular sinuses at the fetomaternal interface [19]. Normally, these invasions are deep in the myometrium and promote the extensive remodeling of the spiral arteries, thus enhancing the blood flow, whereas in the abnormal placenta, the cytotrophoblasts fail to penetrate and cause incomplete remodeling of maternal spiral arteries, leading to placental ischemia and inadequate nutrition [20,21,22]. Furthermore, the arteries become narrow, leading to atherosis caused by the lipid-laden macrophages in the lumen, which cause fibrinoid necrosis of the arteries walls. Moreover, it also affects the decidual vessels and causes decidual vasculopathy (DV), which further compromises the placental blood flow. In PE, the decidual vasculopathy has serious clinical outcomes, such as high systolic BP, severe renal dysfunctions, and perinatal fetal death.

### 4.1. Hypoxia

Recent studies demonstrated the role of increased levels of hypoxia-inducible transcription factors (TFs) in the pathogenesis of PE [23,24]. During early placentation, the gestational sac has low oxygen, which helps trophoblast cells to proliferate. It also connects the blastocytes and plugs the spiral arteries to decidua. Finally, this trophoblastic-spiral plug collapses and forms intervillous space. This sinus enhances the maternal blood flow, producing oxidative stress and facilitating the trophoblast cell differentiation, converting it from proliferative trophoblasts to invasive trophoblasts. These invasive trophoblast cells are responsible for normal placentation, whereas in PE, the cells become deprived of oxygen, and the proliferative trophoblast does not convert into invasive trophoblast cells, hence forming abnormal placenta. [25]. Rajakumar et al. reported that the presence of hypoxia-inducible factors (HIF)-1α and 2α indicates that the cell is deprived of oxygen. A high level of HIF in proliferative trophoblasts has been identified in pre-eclampsia patients [26]. An animal study reported that overexpression of HIF-1α results in hypertension, proteinuria, failure in the trophoblastic invasion, and fetal growth restriction. Canigga et al. proposed that inhibition of HIF-1α can protect the female from maternal syndrome. It further inhibits a potent antiangiogenic factor soluble FMS-like tyrosine kinase 1 (sFLT1) [27,28]. Solhberg et al. reported a higher level of sFLT1 in Swedish women with singleton pregnancies; it reduces the placental perfusion, which is associated with restive fetal growth, maternal abnormalities, low fetal vessel flow [25].

### 4.2. Oxidative Stress

Oxidative stress is also considered one of the major factors in the pathogenesis of pre-eclampsia. An imbalance in reactive oxygen species (ROS) can enhance the production of ROS-inducing enzymes. It may inhibit the Wnt/β-catenin signaling pathways, which play a crucial role in trophoblast invasion. It also increases the production of sFLT1 [29,30,31]. Human studies proposed that abnormalities in the placental antioxidant mechanism can lead to the pathogenesis of PE; for instance, Vaughan et al. reported that the level of superoxide dismutase and glutathione peroxidase decreases in PE patients when compared to normal pregnancies [31]. Recent studies demonstrated that oxidative stress might be derived from mitochondrial stress due to decreased expression of cytochrome C oxidase enzyme [32,33]. This enzyme is a crucial part of the electron transport chain (ETC) in syncytiotrophoblast cells. A decrease in its level has been associated with increased expression of sFLT1 [34]. Furthermore, numerous studies reported that oxidative stress can be triggered by ischemic-reperfusion injury of the endoplasmic reticulum (RT), causing fetal growth restriction and PE [35,36]. It can also cause decidual and cytotrophoblast apoptosis via the activation of unfolded protein response (UPR) [37,38]. In addition, it is found to be responsible for an increase in PERK (PKR-like endoplasmic reticulum kinase), which reduces the translational burden of RT and enhances the proapoptotic TFs, causing PE [38]. Several studies have shown evidence of the role of heme oxygenase (HO) as an important enzyme in the vascular functioning of placenta and fetal development [39,40]. HO-1 is expressed in noninvasive trophoblasts, whereas HO-2 is expressed in spiral arteries, playing a pivotal role in spiral artery invasion. HO-1 is responsible for attenuating the production of superoxide and sFLT-1 in placental vasculature, protecting it from hypoxia-induced changes. Any changes in HO-1 or reduction in its level might lead to placental dysfunction [41,42].

### 4.3. Natural Killer Cell

The evidence of uterine natural killer cells in the pathogenesis of PE has been reported in numerous studies [43,44]. They play roles in abnormal placenta development, spiral artery remodeling, and trophoblastic invasion [45]. These cells also interact at the allogenic fetomaternal cell interface, recognizing the self-major histocompatibility complex (MHCs). Furthermore, it expresses killer cell Ig-like receptors (KIR) in the mother and KIR ligand in the fetus, leading to human leukocyte antigen-C (HLA-C) polymorphism [46]. These KIR and HLA-C polymorphisms may cause abnormalities in the placenta, which can lead to complications such as defective spiral artery remodeling, enhancing the risk of PE [47].

## 5. Stage 2 of Pre-Eclampsia Pathogenesis

Stage 2, also known as “maternal syndrome”, usually occurs in the second or third trimester. It may include an imbalance in several antiangiogenic proteins, inflammatory cytokines, and immune cell alterations.

### 5.1. Elevated Antiangiogenic Proteins

Previous studies reported an elevated level sFLT1 in the placenta of PE patients [48]. sFLT1 is an antiangiogenic factor that is responsible for inhibiting the proangiogenic proteins, vascular endothelial growth factor (VEGF), and placental growth factor (PIGF) [49]. Similarly, soluble endoglin (sEng) is another antiangiogenic protein, also known as a transforming growth factor (TGF-β1) inhibitor [50]. TGF-β is an angiogenic factor, whose mechanism is not well elucidated. sEng, along with sFLT1 induces severe PE, coagulation, liver dysfunctions, nervous disorders, and fetal growth restrictions [51,52]. A study reported that the level of sEng was 4 times higher in the placenta of women with PE than in normal women. Animal model studies reported that sEng reduces the binding of TGF- β1 to its receptor, causing vasodilation. This downregulates the nitric oxide synthase production, which further reduces the endothelial tube formation, affecting angiogenesis [51]. Furthermore, the dysregulation in interleukin-10 (IL-10) can also be the reason for pathogenesis, as it acts as a mitigator of proinflammatory mediators, angiotensin I and II autoantibodies (AT1-AA), endothelin-1 (ET1), and oxidative stress in the placenta [53,54,55].

### 5.2. Response to Proinflammatory Mediators and Immune Cells

The maternal inflammatory response to proinflammatory cytokines is an emerging problem in pregnant women that might lead to the pathogenesis of PE. It is a well-established fact that PE is an inflammatory state. The allogeneic nanovesicles (syncytial knots) that are shed from apoptotic trophoblasts have been identified in the plasma of PE patients [56,57]. Furthermore, sFLT1, endoglin’s exosomes, and peripheral mononuclear cells also initiate inflammatory responses [58,59,60]. Regulatory T cell (Tregs) plays a pivotal role in self-immune response and holds peripheral tolerance against antigens such as autoimmunogens and allergens. It is also important to maintain maternal tolerance for the fetus. It stimulates the production of anti-inflammatory cytokines such as IL-10 and TGF-β [49]. Production of Treg cells in pregnant females leads to normal pregnancy. However, studies have reported that decreased levels of Tregs can cause complications such as miscarriage or pre-eclampsia [61]. Extensive clinical evidence has supported the role of complementary systems in the pathogenesis of pre-eclampsia [62,63]; for instance, hemolysis elevated liver enzyme low platelets (HELLP syndrome) [64] and hemolytic uremic syndrome (aHUS), along with several other findings [65,66]. These disorders develop due to excessive activation of the complement system. An elevated level of C5b-9 has been observed in PE patients. It is a very useful biomarker to differentiate between PE and other hypertensive disorders [63,67]. Other histopathological and immune studies have revealed that the elevated levels of C4d-a and C1q in the kidney glomeruli and placenta of patients may indicate the pathogenesis of PE [68]. Furthermore, evidence of involvement of the renin–angiotensin–aldosterone system in PE pathogenesis has also been reported by Wang et al. and Irani et al., particularly angiotensin II sensitivity [69,70]. This might be due to the presence of AT1 autoantibodies in the serum of PE patients [71]. Numerous studies reported that AT1-AA produces hallmark symptoms of PE, such as vasoconstriction [71], tissue necrosis and umbilical vein cells apoptosis [72], hypercoagulation [72], a decrease in trophoblast invasion [73], and elevated ROS in animal models [74].

## 6. Risk Factors Associated with Pre-Eclampsia

**Figure 2:**
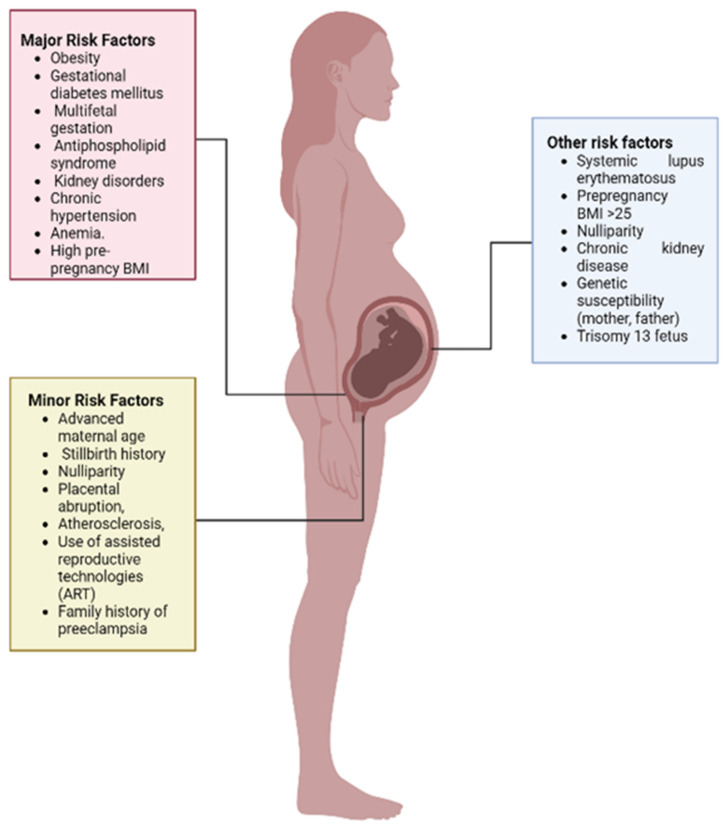
Risk factors of pre-eclampsia differentiated into major, minor, and others. Abbreviations | BMI, body mass index; ART, assisted reproductive technologies.

There are many risk factors associated with pre-eclampsia and extensive studies have been conducted worldwide. Major risk factors include pre-existing disorders (obesity, gestational diabetes mellitus, kidney disorders), multifetal gestation, antiphospholipid syndrome, and most importantly, chronic hypertension. Minor risk factors involve age (young or advanced maternal age), family history of pre-eclampsia, stillbirth history, first pregnancy, nulliparity, placental abruption, atherosclerosis, and use of assisted reproductive technologies (ART) [75,76]. The development of pre-eclampsia also includes some other factors, as shown in Figure 2, that may be influenced by fetal, maternal, or environmental risks or genetic polymorphism.

### 6.1. Pre-Existing Disorders

Evidence has shown that there is an increased prevalence of disorders such as chronic hypertension, diabetes, and obesity, which might lead to PE [77]. Pre-existing diseases are considered to be major risk factors in causing PE. Studies have shown that women with insulin-dependent diabetes are at 4 times more risk of developing PE if it is present before pregnancy [78]. Similarly, pre-existing hypertension can also induce PE pathogenesis. Davies et al. reported that women with hypertension were 12.1% more likely to develop PE when compared to women with no hypertension (0.3%) [61]. Women with kidney disorders were also found to be at higher risk (5.3%) of developing PE than women without it (1.8%). Similarly, some case studies showed that women with PE were more likely to develop autoimmune disorders [79].

### 6.2. Multiple Fetal Gestation

Extensive studies performed on the association of multiple fetal pregnancies and PE reported that women with twins are at a three-times higher risk of developing PE than with a single fetus [78], whereas another study reported that women pregnant with triplets might be at a three-times higher risk when compared to twin pregnancy [80].

### 6.3. Antiphospholipid Syndrome

Antiphospholipid syndrome develops due to the presence of antiphospholipid antibodies (anticardiolipin and lupus anticoagulant antibodies) are considered as the risk factors of PE. Studies reported that the level of antiphospholipid antibodies was found elevated in women with PE [81]. About one-third of women with APS might develop PE during pregnancy. Clark et al. reported that testing of antiphospholipid (aPL) should be performed in women with early onset of severe PE (<34 weeks), eclampsia, and HELLP syndrome. Furthermore, they indicated that APS cannot be considered a primary cause of PE pathogenesis but can be considered a secondary risk factor [82].

### 6.4. Maternal Age

Maternal age is considered one of the risk factors in pre-eclampsia; numerous studies reported that women aged above 40 years are at double risk of developing PE [83,84]. Advanced maternal age is associated with an increased risk of stillbirth, miscarriages, gestational diabetes mellitus (GDM), fetal growth restriction, and C-section [83]. A retrospective study conducted by Khalil et al. reported that advanced maternal age (≥40 years) increases the risk of miscarriage (OR, 1.49, *p* < 0.001), PE (OR, 1.46, *p* < 0.001), GDM (OR, 1.88, *p* < 0.001), and C-section (OR,1.95, *p* < 0.001), but it was not found to cause stillbirth, gestational hypertension (HG), or spontaneous preterm delivery [85].

### 6.5. Nulliparity

Results of a cohort study supported the fact that nulliparity increases the risk of PE up to three times [86]. Furthermore, a case control study reported that women with PE are twice as likely to be nulliparous as women without PE [87]. Shen et al. reported that nulliparous women are at higher risk of developing PE [88]. Another cohort study performed on Chinese and Swedish populations strongly associated nulliparity with severe PE in Swedish women in comparison to Chinese women. They also reported that the association of nulliparity with pre-eclampsia depends on lifestyle and health care [89].

### 6.6. Family History

Family history is always associated with the pathogenesis of many diseases, whether that is hypertension, diabetes, or some autoimmune disorders. Women with such disorders are more prone to develop pre-eclampsia than women without it. There have been studies carried out on the recurrence of PE in families, but its heritable aspects are not yet thoroughly understood [90]. It is estimated that it is 30% to 55% heritable, which may include environmental and genetic factors. A study conducted in Denmark estimated that the maternal family history of PE can increase the risk of PE by almost 115%, with the strongest association of early onset. [91]. A study performed on the Taiwanese population showed that women with a sororal history of PE were at higher risk of developing PE than women with no sororal history [90].

### 6.7. Women with History of PE

Many cohort studies reported that women with PE in the first pregnancy are 7 times more prone to develop PE in the second pregnancy [92,93]. This indicates that women with a history of PE during pregnancy can develop it in the future as well. A study indicated that women with a history of PE can also develop cardiovascular disorders [94].

### 6.8. Placental Abruption

PE is classified as one of the three ischemic placental diseases (IPD), as its pathogenesis includes placental abruption and intrauterine growth restriction, which leads to uteroplacental underperfusion [95]. The prevalence of placental abruption in the US is 2.3% among preterm deliveries and 0.3% among term deliveries [96]. Samantha et al. reported that preterm placental abruption increases the risk of PE 2-fold (OR 2.2, CI 1.5,3.3). Furthermore, they also found out that this association is prevalent among women with a history of PE [97].

### 6.9. Paternal Risk

An interesting paternal risk factor hypothesized by Dekker et al. is the “dangerous father”. It was proposed that a father whose partner previously had pre-eclampsia is more likely to cause PE in the next pregnancy in the same partner or new one [98]. The occurrence of pre-eclampsia in young women is often attributed to the immune mechanism. It occurs due to the tolerance developed by the maternal immune system to the paternal exposure to seminal fluid and/or sperm. The longer the exposure to semen, the lesser the risk of PE will be [99].

## 7. Genetic Polymorphism and Its Association with Pre-Eclampsia

Genetic susceptibility is considered a relatively rare risk factor; however, it is one of the extensively studied domains for PE [76]. These alterations in genes cause changes in the structure of the proteins, leading to impaired protein functioning.

Based on prior biological evidence, more than 70 candidate genes are associated with the pathogenesis of PE. These genes can be separated depending on proposed pathophysiological mechanisms, for instance, oxidative stress, vasoconstriction, thrombocytopenia, lipid metabolism, endothelial injury, and immunological dysfunction. Despite extensive studies, no gene is found to be a primary cause of PE. However, there is evidence of polygenic association of PE [100]. Here, we discuss candidate genes that are associated with PE.

### 7.1. FMS-Like Tyrosine Kinase 1

FMS-like tyrosine kinase 1 (FLT1) gene polymorphism has been associated with PE in numerous studies. The gene is located in the 13q12 region of the chromosome and has 29 introns and 30 exons. FMS-like tyrosine kinase (Flt-1) is one of the receptors of VEGF, also known as VEGF 1, having negative and positive activity on vascular development. VEGF is the most important angiogenic factor, which plays a pivotal role in angiogenesis, such as endothelial cell proliferation, migration, and regulation of permeability of vasculature. A study performed on mice embryos reported that FLT-1 is responsible for inhibiting vascular proliferation, whereas in adults, FLT-1 was responsible for stimulation of mild angiogenesis. Hence, it is reported that it not only regulates the balance in angiogenesis in the placenta but also helps in the proliferation of trophoblast cells [101]. In 2017, a genome-wide study performed on the FLT1 gene showed that polymorphism at a locus (rs4769613) has pathogenetic effects in PE, as shown in Table 2. It particularly exerted harmful effects on the fetal genome. A placental isoform sFLT-1, which was found to be associated (*p* = 54 × 10^−11^; 310,238 controls and 4380 cases) with the pathogenesis of pre-eclampsia, is an antiangiogenic protein. Another variant, rs12050029, was also found to be associated with PE, regardless of the neighboring locus (rs4769613) [102].

### 7.2. Endothelial NOS Gene (eNOS)

Endothelial nitric oxide synthase (eNOS) plays a crucial role in the regulation of endothelial functioning and regulated vascular tone and homeostasis. eNOS is the main regulator of vascular tone, which is regulated by post-translational modifications and protein interactions. Any changes in the eNOS gene can disrupt the formation of NO, which can lead to hypertensive disorders or cardiovascular disorders. eNOS is located on chromosome 7q35-36. It has 26 exons and 25 introns with 120 amino acids and 135 KDa protein.

Procopciuc et al. reported the relationship of the eNOS gene with the pathogenesis of PE. The results showed the significant association of eNOS (Glu298Asp) polymorphism in the development of PE. eNOS is responsible for the production of nitric oxide (NO), which acts as a vasodilator. Any change occurring in this gene can affect the production of NO, which can lead to hypertension, coronary artery disorder, and PE. Furthermore, another study reported the SNP polymorphism in eNOS, also caused endothelial dysfunction, which can increase oxidative stress [10]. Another study performed on the Turkish population indicated that eNOS gene (Glu298Asp) polymorphism was more prevalent in PE patients and may lead to eclampsia [103]. These results were in contrast to the previous studies conducted by Fatini et al., Kim et al., and Singh et al., which showed no association of eNOS with the development of PE [104,105,106]. More studies need to be performed on other variants to evaluate the association of eNOS polymorphism with PE.

### 7.3. Angiotensin-Converting Enzyme Gene

Polymorphism in the angiotensin-converting enzyme gene (ACE) gene (rs4343) can disrupt the functioning of the RAS (renin–angiotensin system), which is responsible for homeostasis. The level of ACE in the tissue and serum is very important in maintaining blood pressure. The gene is located on chromosome 17 with 25 exons and 24 introns. The ACE gene can lead to increased vasoconstriction and decrease the level of vasodilators, causing hypertension. Abedin Do et al. reported the association of polymorphism in the rs4343 variant of the ACE gene to PE [107]. Moreover, previous studies also demonstrated its role in CAD, migraine, and ventricular hypertrophy. In another study, Zhu et al. reported that the role of the rs4343 G allele variant was responsible for increased systolic and diastolic blood pressure. The presence of the G allele was also studied in depression patients [108]. Numerous case control studies and meta-analyses have shown the association between the ACE gene (I/D) and PE (Table 2).

### 7.4. Interleukin-10 Gene

Daher et al. reported that increased levels of interleukin-10 (IL-10) in pregnant women lead to a successful pregnancy, whereas lower production can cause PE. IL-10 is located at 1q31-32 on chromosome 1. IL-10-(1082) gene polymorphism shows a strong association with PE, particularly in black people. This shows that ethnicity has a strong influence on the incidence of disease [109]. A case control study performed by Limeng Song reported that the genotype -1082A/G (rs1800896) was not found to be associated with pre-eclampsia, whereas genotype -592A/C(rs18000872) was significantly associated (CC genotype and AC + CC) to the onset of PE [110]. Table 2 demonstrates the other variants responsible for PE pathogenesis in various ethnic groups.

### 7.5. Human Leukocyte Antigen-G

HLA-G plays a crucial role in the maintenance of the maternal immune response throughout pregnancy and alleviates the immunogenic reactions during pregnancy [111,112]. It is expressed by the extravillous trophoblast cells in the placenta and modulates the immune responses during vascular remodeling [113]. Tan et al. reported a significant association between HLA-G-G*0106 in the fetus and the development of PE. It increases the prevalence of PE in multigravidas, which does not carry the G allele of HLA-G-G*0106, due to an increase in the alloimmune response. Human leukocyte antigen-G is present in the trophoblast cells of the fetus; its major role is to inhibit the trans-endothelial migration of NK cells through the placenta. They also reported the mismatched genotype of HLA-G in PE mother and fetus [114]. Another study performed on three SNPs, HLA-G (rs29794467, rs29796376, rs29799440), showed that only genotype rs29799440 had a low association in PE patients containing the CC genotype (*p* = 0.047), while there was no association was found in individuals having the TT and CT genotype [115]. Other variants are also discussed in Table 2, which shows the positive association in PE pathogenesis.

### 7.6. Prostatin Gene

Prostatin is responsible for tumor formation, invasion, and metastasis. The mechanism of trophoblast invasion is quite similar to tumor formation, and prostatin is responsible for its formation. Moreover, prostatin also activates the epithelial sodium channel and affects blood pressure. However, the mechanism of prostatin’s polymorphism in PE pathogenesis is still unclear. A study conducted on Chinese populations reported that the TC or CC genotype at rs12597511 was responsible for multiple complications, such as liver dysfunction, neonatal intracranial hemorrhages, asphyxia in neonates, and perinatal mortality. Furthermore, a study reported that the TC or CC genotype was also found to be significant in severe PE patients (OR = 1.049, *p* = 0.021; OR = 1.031, *p* = 0.013; OR = 0.733, *p* = 0.019) [116]. Another study supported the results that TC + CC genotypes is associated with severe PE patients (*p* = 0.001) [117]. Ejaz and coworkers also investigated the association of prostatin gene variants with PE. They reported that the C genotype of (rs12597511) was significant in PE patients. Therefore, the C allele polymorph might be a potential risk to PE pathogenesis [118].

### 7.7. CXC Chemokine Receptor 2 Gene (CXCR2)

CXCR2 is part of the chemokine superfamily that is named based on the position of cysteine amino acid in the protein [119]. It belongs to the CXC family of chemokine receptors. CXCR2 plays an important role in inflammation [120], oxidative stress [121], vascularization [122], tumorigenesis [123], and immunity [124]. Previous studies reported that CXCR2 interferes with maternal–fetal interface immunity and decidual spiral artery remodeling; therefore, it might induce PE pathogenesis [124,125]. Recent studies showed that low levels of CXCR2 in the placentae may contribute to the pathogenesis of PE via downregulation of matrix metalloproteinase-2 (MMP-2) and matrix metalloproteinase-9 (MMP-9), which impairs the invasion of the trophoblast [126]. A study performed by Chen et al. in Han Chinese populations on the CXCR2 genes rs1126579 and rs2230054 supported the above-mentioned theories. It was found that the rs1126579 variant was strongly associated with the development of PE in Han Chinese women [127].

### 7.8. Forkhead Box P3

The forkhead box P3 (FOXP3) gene is located on the X chromosome, which is responsible for the formation of FOXP3 protein. It is responsible for regulating the immune system by binding with the specific DNA region. The FOXP3 gene, also known as forkhead/winged-helix transcription factor, is mapped to chromosome Xp11-23 and is important for the proper function and development of Treg cells. The polymorphism in the FOXP3 gene leads to the suppression of Treg cells. Studies reported the association of FOXP3 variants/polymorphs with various disorders such as systemic lupus erythematosus (SLE), atherosclerosis, autoimmune thyroid disease (AITDS), and PE.

Jiying et al. investigated the association of single-nucleotide polymorphism (SNPs) of FOXP3 with different genotypes in the Chinese population. They reported that the genotype variants of FOXP3; rs3761549 C > T, rs2232365A > 6, rs2280883T > C and rs3761548C > T of healthy pregnant women (controls = 243) and PE patients (n = 203). The study reported that FOXP3 rs2232365 has more association with severe PE patients and less association with mild pre-eclampsia patients [128]. However, a study performed on Iranian women by Gholami et al. on FOXP3 reported conflicting results. They suggested that the allelic frequency of rs2232365 was not associated with PE, under codominance (OR = 0.49, 95% CI, *p*= 0.027) nor under overdominance (OR = 0.54, 95% CI, *p*= 0.02), whereas rs3761548 showed the risk of PE in the recessive model (OR 2.05, 95% CI, *p* = 0.025). Furthermore, they found no mutation in exon 2 of FOXP3 [129].

### 7.9. Inhibin Beta B Gene

The inhibin beta B gene (INHBB) protein is the beta subunit of inhibin, which is a pituitary follicle stimulating hormone inhibitor. There has been evidence of its polymorphism playing a role in PE pathogenesis. A study performed on three SNPs (rs12711941, rs7576192, and rs7579169) in the Chinese Han population suggested that rs7579165 CC genotypes and GG haplotypes were significantly associated with being risk factors for developing PE, particularly in late-onset and multifetus pregnancy [130].

### 7.10. Other Candidate Genes

Numerous studies reported the presence of proinflammatory cytokines in the amniotic fluid of PE patients, for instance, IL-2, IFN-c, TNF- α, and IL-6. These cytokines have deleterious effects on pregnancy, whereas IL10 and TGF-b were reported to have a positive impact on the promotion of pregnancy. Various other single-nucleotide polymorphism (SNPs) studies were reported by numerous scientists around the world on different ethnicities and showed association with PE pathogenesis, for instance, GSTP1, IL-279, IL-4 C-590T, IL-4 VNTR RP2 allele, IL-1α (rs17561 TT and GT), IFN-γ + 874T/T, VEGF-C rs7664413, CYP24A1, and CYP27B1 [3]. The summary of candidate genes associated with the pathogenesis of pre-eclampsia is shown in Table 2.

**Table 2:** Candidate genes associated with the pathogenesis of pre-eclampsia.

Candidate Gene	Polymorphism	Study Type	Population	Total Participants		Association with PE	Ref.
				PE	Control		
*FLT1* gene	Locus (rs4769613) T/C	Meta-analysis (Data were collected from 13 case control studies)	UK and Iceland	4380	310,238	Positive association was found	[131]
		Meta-analysis (Data were collected from 8 case control studies)	Estonia	96	2001	Positive association was found	[131]
	rs722503	Case control study	Iranian	204	191	Positive association found	[132]
***eNOS***	Glu298Asp	Case control study	Romanian	69	94	Positive association found	[10]
		Meta-analysis (Data were collected from 15 case control studies)	Multiethnic group	1610	2975	Mild association was found	[133]
		Case study	Chinese	92	256	Positive association was found	[134]
	-786 T/C	Case control study	Serbian	50	50	Positive association was found	[135]
	intron 4 (VNTR4b/a)	Case control study	Serbian	50	50	Positive association was found	[135]
	G894T	Meta-analysis	Multiethnic group	2450	4927	Positive association was found	[136]
*ACE* gene	I/D intron (D-allele)	Case control study	Iran	165	131	Positive association was found	[107]
		Case control study	Mexican	66	37	Positive association was found	[137]
		Case control study	Pakistan	200	200	Positive association was found	[138]
		Case control study	Korean	104	114	No association was found	[139]
		Meta-analysis (data were collected from 30 case studies)	Asians and Caucasian	3184	3912	No association was found for Asians and positive association was found in Caucasians	[140]
		Meta-analysis	Columbian	665	1046	Positive association was found	[141]
		Meta-analysis (data were collected from 11 case studies)	Chinese	800	949	Positive association was found	[142]
*IL10* (Interleukin 10)	-592A/C	Case control study	Chinese	155	201	Positive association was found	
		Meta-analysis (Data were collected from 11 case control studies)	Asian and South American	1861	3632	Positive association was found	[143]
		Case control study	Indian	120	120	Positive association was found	[144]
	819T/C	Case control	Chinese	177	182	Positive association was found	[145]
		Case control study	Indian	120	120	Positive association was found	[144]
		Meta-analysis (Data were collected from 11 case control studies)	Multiethnic	1861	3632	Positive association was found in Asian and South American populations	[143]
	1082-G/G	Case control study	Brazilian (white and nonwhite women)	151	189	Mild association was found in white women	[109]
		Case control study	Indian	120	120	No significance was found	[144]
*HLA-G*	-201AA	Case control study	Multiethnic	116	130	Positive association was found	[146]
	14 BP (I/D) (rs66554220)	Meta-analysis (11 studies)	European Caucasian	240	158	Positive association was found	[147]
	14 BP (I/D) (rs1704, +2961 − 2974)	Case control study	Norway, Netherlands, and UK	83	83	Positive association was found	[148]
	rs29799440 (CT/TT)	Case control study	Chinese Han	51	48	Positive association was found	[115]
	G*1060	Case control study	Southeast Asian	83	240	Positive association was found	[114]
*Prostatin* gene	rs12597511 (TC/CC)	Case control study	Chinese	179	222	Positive association was found	[116]
		Case control study	Pakistani	76	74	Positive association was found	[118]
*Foxp3*	rs2232365 (A/G)	Case control study	Chinese Han	203	234	Positive association was found	[128]
		Meta-analysis (Data were collected from 7 case control studies)	Multiethnic	784	1415	Positive association was found	[15]
		Case control study	Iranian	133	143	Positive association was found	[129]
		Meta-analysis (6 studies)	Multiethnic	1231	1384	Positive association was found	[149]
	rs3761548	Case control study	Iranian	133	143	Positive association was found	[129]
		Meta-analysis (8 studies)	Multiethnic	784	1631	Positive association was found	[15]
		Meta-analysis	Multiethnic	1023	987	Positive association in Asian Population	[150]
		Meta-analysis (7 studies)	Multiethnic	1513	1600	Positive association was found	[149]
*INHBB*	rs7579169 (CC) genotype	Case control study	Chinese	181	203	Positive association was found	[130]

Abbreviations|FLT1: FMS-like tyrosine kinase 1; eNOS: endothelial NOS; ACE: angiotensin-converting enzyme; IL-10: interleukin-10; HLA-G: human leukocyte antigen-G; CXCR2: CXC chemokine receptor 2; FOXP3: forkhead box P3; INHBB: inhibin beta B.

## 8. Conclusions and Future Recommendations

Being a disease of theories, PE has always been one of the primary reasons for fetomaternal morbidity and mortality worldwide. Extensive studies performed on PE have proposed numerous mechanisms of its pathogenesis, for instance, spiral arteries, endothelial dysfunction, oxidative stress, placental perfusion, and altered immune response. Furthermore, many maternal, fetal, environmental, and genetic risk factors are involved in the development of this hypertensive disorder. Evidence shows that sFLT1 (potent antiangiogenic protein) affects placental perfusion, and HLA-C and KIR cause defective spiral artery remodeling. Studies also reported that the inhibition of VEGF, PIGF, IL-10, TFG- β, Tregs, and production of AT1-AA can lead to PE. Genetic polymorphism plays a crucial role in the progression of PE; all these genes—FLT1, eNOS, ACE, IL10, HLA-G, Prostatin, CXCR2, FOXP3, INHBB—were found to be significantly associated with PE pathogenesis. Despite these studies, the etiology of PE is still fuzzy. More case control and genome-wide association studies (GWAS) need to be carried out in order to unravel the primary causes involved in the PE pathogenesis. These studies will promote the individualization of treatment, the discovery of new targets, and the removal of clinical trials and errors.

## Figures and Tables

**Table 1:** Pre-eclampsia biomarkers according to ACOG.

Pre-Eclampsia	
Blood pressure	Systolic BP ≥ 140 mm Hg Diastolic BP ≥ 90 mm Hg
Proteinuria	Level of protein in urine ≥ 300 mg/ 24 h Protein/urea creatinine ≥ 0.3 or more Dipstick test reading = 2+
In absence of proteinuria	Thrombocytopenia: platelet count less than 100 × 10^−9^/L Impaired kidneys: serum creatinine ≥ 1.1 mg/dL or double Impaired liver: elevated levels of transaminases to almost double Lungs: pulmonary edema Headache, impaired vision
**Severe Pre-Eclampsia**	
Blood pressure	Systolic BP ≥ 160 mm Hg Diastolic BP ≥ 110 mm Hg
Thrombocytopenia	Thrombocytopenia: platelet count less than 100 × 10^−9^/L
Liver dysfunction	Elevated level of transaminases in blood more than twice than normal concentration Epigastric pain unresponsive to medication
Renal dysfunction	Serum creatinine concentration greater than 1.1 mg/dL or double
Other	Lungs: pulmonary edema Headache unresponsive to medication Visual disturbance

## Data Availability

Not applicable.
